# Drivers’ Braking Behavior Affected by Cognitive Distractions: An Experimental Investigation with a Virtual Car Simulator

**DOI:** 10.3390/bs10100150

**Published:** 2020-10-01

**Authors:** Nicola Baldo, Andrea Marini, Matteo Miani

**Affiliations:** 1 Polytechnic Department of Engineering and Architecture (DPIA), University of Udine, Via del Cotonificio 114, 33100 Udine, Italy; matteo.miani@phd.units.it; 2 Department of Languages and Literatures, Communication, Education and Society (DILL), University of Udine, Via Margreth 3, 33100 Udine, Italy; andrea.marini@uniud.it; 3 Claudiana—Landesfachhochschule für Gesundheitsberufe, I-39100 Bolzano, Italy

**Keywords:** pedestrian crossing, driver’s behavior, car driving simulator, cognitive distractions, human factors, speed reduction measures, earphones

## Abstract

In this study, a cohort of 78 university students performed a driving experience in a virtual urban scenario, by means of a car driving simulator, to examine effects of a planned hands-free mobile phone conversation on young drivers’ braking behaviors. To this aim, a control group was left free to drive without any imposed cognitive task. An experimental group faced the same scenario while engaged in a phone call. The conversation via earphones was arranged to diminish the amount of cognitive resources allocated to the driving task. For both groups, the analyses focused on the moment at which a child entered a pedestrian crossing from a sidewalk. The results of a mixed two-way ANOVA showed the presence of a significant difference for distracted and non-distracted drivers with the absence of gender-related differences across the two groups. Distracted participants assumed lower initial speeds, took the first action to stop at shorter distances from the zebra crossing, and had more difficulty in keeping speed variations under control. These findings suggest that the distraction induced by the use of earphones may induce risk compensation behaviors and delay pedestrian perception. Moreover, the effects on the participants’ braking behavior suggest that the procedure adopted to increase cognitive load, based on a story retelling, is an effective method to analyze the impact of hands-free cellphone use on driving skills in a car simulation experiment.

## 1. Introduction

Driving is a complex activity that requires the integration of both subjective (e.g., driver’s health state, experience, concentration level) and objective (e.g., road condition) variables. Drivers need to constantly monitor the road [1,2] as the information provided by the road environment is pivotal to avoid dangerous behaviors [3,4,5].

Road safety is significantly affected by the concentration levels of drivers. Indeed, they are often distracted by a number of events. They may be mind-wandering or engaged in a conversation with a passenger or a distant friend through their mobile phones. Such events might significantly lower the cognitive resources employed to monitor the road and induce risky behaviors. For instance, the National Highway Transportation Safety Administration (NHTSA) estimated that in 2018 9.7% of drivers in the United States used a mobile phone while driving. In particular, mobile phone use is most frequent among female drivers below 24 years of age [6], with an increasing percentage of drivers using hands-free mobile phones [7]. Furthermore, White et al. [8] showed that 43% of a cohort of 796 drivers in Australia answered calls daily, 36% of them made calls, 27% read text messages, while 18% even sent them. Similarly, Atwood et al. [9] observed an average of 1.6 texts and 1.2 calls per hour of driving by collecting the text and call records of 557 US drivers involved in a three-year naturalistic driving study.

Such distractions may significantly affect drivers’ performance in different ways. For example, they may determine a reduction in speed control, difficulties in maintaining the appropriate trajectories, and even increase their reaction times in responding to hazards [10,11,12,13,14]. This is likely determined by a reduction of the cognitive resources assigned to the driving task [15] as the distraction induced by the use of a cellphone significantly alters the brain activity associated with driving [16]. Therefore, the increased cognitive load related to a telephone call reduces the attention to the road inducing an “inattentional blindness” [17]: the distracted driver’s brain receives the visual input regarding the road environment but does not process such information at a conscious level. This means that the driver is “blind” to such information even if (s)he saw it. Finally, Amado and Ulupınar [18] observed the negative effects of conversation with a remote person or an in-vehicle person on the driver’s attention level and hazard perception skills, showing that these effects did not depend on conversation type (remote/in-person).

Obviously, these considerations pose serious safety problems that need to be considered in experimental research activities. Among the research methods that have contributed to the understanding of crash-relevant behaviors and the identification of safety-critical events, Naturalistic Driving (ND) studies play a fundamental role [19]. In ND studies, recording equipment (including small video cameras and sensors) is installed in participating volunteer drivers’ cars and, for several months to several years, these devices register the vehicle’s speed, acceleration/deceleration and direction, the driver’s gaze shifts, head and hand movements, as well as road, traffic, and weather characteristics. The recorded data are used to identify, by means of kinematic triggers, the safety-critical events, such as crashes and near-miss incidents, with the aim of evaluating risk factors during the seconds leading up to a crash and determining the risks associated with various observed drivers’ behaviors (e.g. phoning, talking with passengers on board, speeding). The relative risk of a safety-critical event caused by a given behavior can be estimated by comparing the occurrence of such risk-related behavior at the time of the crash or near-miss incident with the proportion of the normal-driving time where the selected behavior occurs (also called the “prevalence” of the potential risk-related behavior). In particular, Dingus et al. [20] reported that almost 90% of the crash events recorded in the National Academy of Science-sponsored ND studies were determined by driver-related factors (i.e., errors, impairments, fatigue, and distraction) and, especially, by the visual-manual secondary task distraction resulting from the use of a handheld mobile phone (i.e., dialing, talking, texting, reaching for the phone or browsing on the phone). Such behaviors increase the risk of a car accident by 3.6 times. In addition, Guo et al. [21] showed that the risk of secondary-task engagement is higher for younger drivers compared with middle-aged drivers. A recent study [9] showed that cellphone texting and calling are still common while driving and drivers who have high prevalence of cellphone use have higher crash rates: with respect to the average cellphone use of 557 US drivers, the crash rate increased for every additional text per day and for every text per hour of driving. On the contrary, Dingus et al. [22] showed that, even if significantly affecting driving performance, distraction (i.e., talking/singing alone in the vehicle, conversing on a handheld or hands-free cellphone, and conversing with a passenger) did not increase the odds ratio of a car crash.

Naturalistic studies have provided relevant information about the prevalence of various risk-related behaviors. Nonetheless, the use of driving simulators can be informative in research activities aimed at assessing the common effects of such behaviors on the road users’ driving performance and at improving road geometric design criteria [23,24]. As for the use of mobile phones, in Burns et al. [25] cellphone conversations affected speed control and response to traffic signals more than having a blood alcohol level of 80 mg/100 mL. Furthermore, Rakauskas et al. [26] reported that the cognitive distraction induced by mobile phone use may cause slower driving speeds with significant fluctuations. The reduction in speed has been interpreted as a risk compensatory effect for the increased mental workload [27]. By controlling different driving conditions (i.e., free flow, stable flow, and oversaturation) with a driving simulator, Stavrinos et al. [28] showed that distracted driving, particularly texting, may lead to risky behaviors with a negative impact on traffic flow. Similarly, in Haque and Washington [29], cognitive distractions significantly compromised reaction times in a cohort of young drivers while facing a traffic event (i.e., a pedestrian entering a zebra crossing from a sidewalk). Unfortunately, to the best of our knowledge, potential gender-related effects have been only scarcely considered in driving simulator studies [30].

## 2. Research Topic and Scope

Encounters between cars and pedestrians at the zebra crossing determine a critical situation in which the safety of the pedestrian mainly depends on the driver’s attention to the road environment, driving control, and speed [31]. The pedestrian’s decisions are influenced by the perceived dynamic parameters and distance of the vehicle, which define the driver’s arrival time at the crosswalk. Depending on such a time, the driver decides whether to deny or give priority to the pedestrian [32]. Therefore, the time taken by the vehicle to reach the crosswalk as soon as the pedestrian arrives at the edge of the curb is a relevant variable in the description of the pedestrian - driver interaction. Such a variable, called Time-To-Zebra ($TTZ_{d}$) in the literature [31], also determines the time available for the driver to react to the pedestrian presence, as it is defined by the ratio between the vehicle’s distance and speed from the crosswalk when the pedestrian is about to cross and the driver perceives his/her presence.

Some studies have focused on the behavior of drivers while approaching a zebra crossing [30,31,32,33,34]. However, the speed profiles of drivers engaged in taxing phone conversations have been insufficiently explored so far. This gap in research is particularly relevant given that mobile phone use while driving appears to be more widespread among young and less experienced drivers, who remain over-represented in road accident statistics [35].

By using a motion-base driving simulator, the current investigation focused on the effects of a taxing hands-free mobile phone conversation on young drivers’ braking maneuvers when a pedestrian enters a zebra crossing. In particular, two groups of young drivers were selected. A control group was formed by individuals driving in an urban scenario with no distraction. An experimental group included participants who were asked to drive while engaged in narrative discourse retelling task during a mobile-phone conversation. The analyses focused on the moment in which the drivers in the two groups reached a zebra crossing with a pedestrian crossing it. This scenario was reproduced in the present study by adopting a $TTZ_{p}=3\;s$ (i.e., the pedestrian started crossing the road when the ratio between the distance from the crosswalk and the vehicle speed was 3 s), as suggested by the reviewed literature [33], with the aim of forcing the vehicle to stop. It is worth pointing out rigorously that $TTZ_{d}$ and $TTZ_{p}$ do not coincide because the driver changes his/her driving behavior as soon as (s)he perceives a possible conflict with the pedestrian (i.e., usually before the pedestrian starts crossing), who in this experiment did not appear suddenly but was clearly visible about 200 m from the crosswalk. Therefore, the $TTZ_{d}$ represents the actual interaction condition: such variable is linked to the kinematic parameters of the vehicle at the moment the driver reacts to the pedestrian presence and not at the moment the pedestrian starts to cross [33,36]. The motivation behind accounting for this variable was that it represents an indicator of pedestrian safety at a crosswalk. Furthermore, we also analyzed the potential effect of gender by controlling for gender-related effects within the two groups of participants.

## 3. Materials and Methods

### 3.1. Participants

Eighty university students were recruited for the experiment. Two participants were excluded from the experiment as they experienced motion sickness and simulator discomfort. These young drivers were recruited by means of a request for participation sent to the university e-mail addresses of students, enrolled in different (Civil Engineering, Agricultural Science, Legal Services, and Public Relations) bachelor degree courses of the University of Udine. Inclusion criteria were an age between 20 and 30 years, having a valid European driving license, and the absence of any illness that could compromise the driving activity. Subjects participated on a voluntary basis with no monetary reward. All of them filled in a questionnaire [29] on their demographics and driving behavior. Their non-verbal intellective quotient (IQ) was indirectly assessed by administering the Raven’s Colored Progressive Matrices [37].

All participants gave their informed consent for inclusion before participating in the study. The study was conducted in accordance with the Declaration of Helsinki, and the protocol was approved by the Local Ethics Committee of Udine’s University (Progetto_Guida).

Participants formed a control and an experimental group. The control group was formed by 15 males and 11 females who were asked to drive in a simulated urban scenario with no distraction. Their mean ages were 23.5 (SD 1.55) for males and 22.6 (SD 1.96) years for females. Their non-verbal IQ levels were 33.7 (SD 1.63) for males and 32.3 (SD 3.06) for females. The experimental group included 30 males and 22 females who were asked to drive while engaged in narrative discourse retelling task during a hands-free mobile phone conversation. Their mean ages were 24.4 years (SD 2.14) and 23.8 (SD 3.36) years, respectively. The average non-verbal IQs for males and females in the experimental group were 33.9 (SD 1.72) and 33.2 (SD 2.24), respectively.

The two groups were balanced with respect to age and IQ level. Furthermore, 81% of the participants had held a driving license for at least three years. A total of 53% of participants drove less than 10,000 km per year; 41% drove about 10,000–20,000 km per year; the remainder drove more than 20,000 km in a typical year. None of the participants had received an infraction notice for red light running (Art. 146), use of the mobile phone (Art. 173) or driving under the influence of alcohol and/or drugs (Art. 186-187). However, 21% of them had been involved in a road accident over the past three years and another 12% had received an infringement notice for exceeding speed limits (Art. 142 of the Italian Road Traffic Code). Finally, many of them (72 %) declared that they use their mobile phone while driving (please, refer to Table 1 for a complete overview regarding their cellphone use while driving).

### 3.2. AutoSim 1000-M Driving Simulator

**Figure 1:**
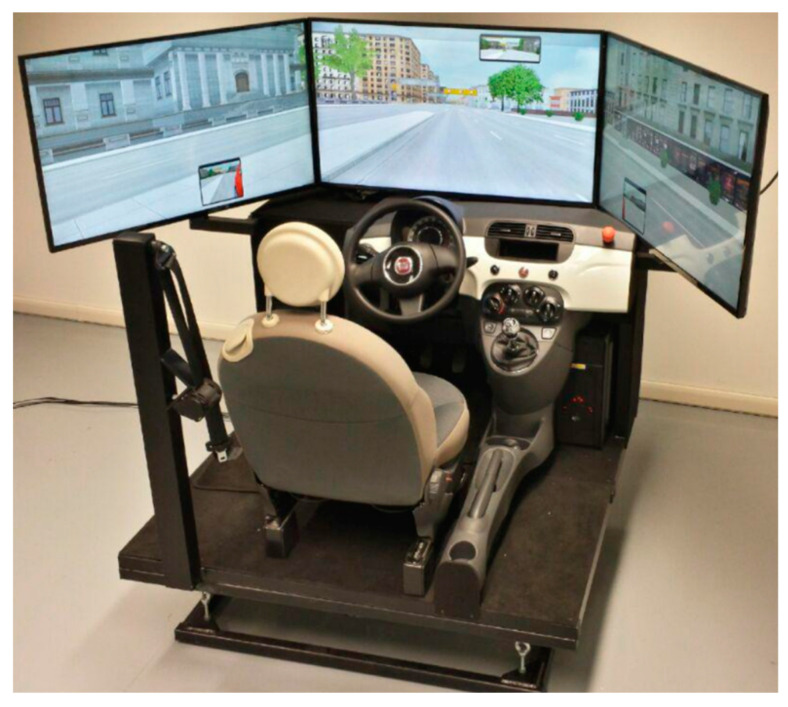
The AutoSim 1000-M car simulator.

The experiment took place at the Roads Laboratory of the Department of Civil-Environmental Engineering and Architecture of the University of Udine. Driving was simulated by using the AutoSim 1000-M car simulator (Figure 1). The simulator cabin, composed of real car parts (the same interior equipment of a Fiat 500), is mounted on a two-degree of freedom motion system to reproduce the rolls and pitches of the vehicle in the virtual road environment. The combination of these rotations, as well as the steering force feedback, provide the tested driver with partially realistic driving sensations. In front of the driver’s seat and above the dashboard, three Philips 43-inch LCD screens, connected to two top-of-the-range PCs with Nvidia GTX graphics boards, allow the road scenario to be visually reproduced with a 180° field of view. A HiFi sound system with 3D and doppler effect simulates the noise of the vehicle and the driving environment. Different vehicle types are individually configurable on all relevant parameters (engine power, transmission, physics, etc.) and such information is transmitted to the hardware interfaces (steering wheel, pedals, gear lever and handbrake). During the simulation, many dynamic parameters describing the driver’s behavior, as well as the driver’s actions on the brake pedal, accelerator and steering wheel, can be recorded with spatial or temporal intervals.

### 3.3. Experimental Procedure and Virtual Road Scenarios

This study used two road scenarios that have been simulated in a virtual environment by the Norwegian company AutoSim. Such scenarios reproduce some Norwegian localities and urban districts of the City of Tromsø. In particular, a sub-urban scenario (total driving time of 5 min), with geometric features suitable for the purposes of this study, was chosen to train the participants of both groups to use the driving simulator (usage of gearshift, steering wheel, clutch, accelerator and brake). A second typically urban scenario was engaged for the experimental driving condition, lasting about 15 min. The simulated urban environment was characterized by numerous traffic light intersections, rectilinear short development roads, sharp curves (90°), pedestrian zebra crossings. In the present study the traffic flow in the vehicle lane was not implemented in the scenario, to avoid any influences of the traffic on the driver behavior. The speed limit in the city was mostly 50 km/h, whereas the speed limit in the sub-urban environment varied between 50 and 60 km/h. In both scenarios, the route that participants were required to follow was shown by green arrows that appeared on the central screen. In order to restore psychological conditions similar to those at the beginning of the test and to limit the possible habituation or fatigue of participants, a 5-min break was inserted between the training and the experimental driving, during which participants were asked to fill in a questionnaire [29] about their driving styles and daily use of the mobile phone (even while driving). At the end of the experimental scenario, the participants of both groups completed a second questionnaire on the experience perceived in the virtual environment.

**Figure 2:**
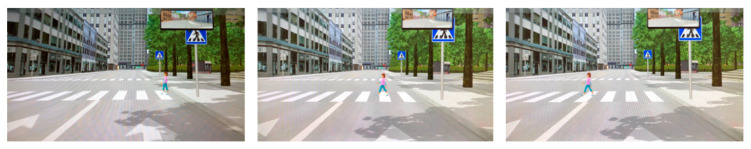
Crosswalk scenario in the driving simulator.

While driving in the experimental scenario, the participants of both groups experienced a traffic event: a girl crossed the road on the pedestrian crossings, starting from the sidewalk. Figure 2 shows the driver’s view of the pedestrian scenario as represented in the driving simulation. This traffic event took place on a four-lane road with two lanes in each direction separated by a continuous center line, where the speed limit was 50 km/h. The crosswalk has been designed by placing appropriate markings and traffic signs for pedestrian crossing according to Norwegian road standards. The traffic event was scripted so that the pedestrian would start moving from a sidewalk to the zebra crossing when the driven car was at $TTZ_{p}=3\;s$ from the crosswalk itself, at a speed of 1.4 m/s in line with the reviewed literature [34]. Although the pedestrian scenario originated from the drivers’ peripheral vision, the drivers had a clear view to the pedestrian and the zebra crossing from 200 m in advance the crosswalk, where a red traffic light forced the participants of both groups to stop.

The control group drove through the experimental scenario without any imposed cognitive task. In this way, data were obtained in reference to the driving behavior under conditions of normal attention on the road (with expected fluctuations of attention levels in monotonous routes). These data included: position of the vehicle on the roadway; operating speeds; accelerations and decelerations.

The experimental group was asked to drive while making a phone call that was planned to diminish the amount of cognitive resources allocated to the driving experience. Specifically, during the 5-min break between the training and the experimental driving conditions, the participants in the experimental group were shown one of three cartoon-picture stories made of six images each (the Flower Pot story [38]; the Nest story [39]; the Quarrel story [40]). The stories were balanced for the number of concepts, words and sentences they might elicit. The order of administration of these stories was rotated from participant to participant in order to reduce a possible story bias.

The stories were shown to the participants on a PC turned towards them, so that the examiner could claim not to know its content. In this way, the possible effect of sharing with the referent was minimized. Participants were asked to mentally imagine the story reported in the stimulus figures and not to report it at that moment. The examiner called each participant in the experimental group prior to the urban drive and a single continuous call occupied both parties until the end of the drive. For 10 min, participants were left free to drive through the urban environment. At the stroke of the tenth minute, the experimenter asked the drivers, connected to their mobile phones by earphones, to tell the story they had previously seen. During the story retelling (whose duration was approximately of 2 min), the drivers in the experimental group suddenly saw the girl entering the zebra crossing. Finally, the last few minutes of driving were free of distractions.

### 3.4. Driving Data

The impact of mobile phone distraction and the potential effect of gender on drivers’ behavior were assessed considering the vehicle speed and its variations nearby the pedestrian crossing for each group of drivers. As we did not have cameras that could monitor the participants’ eye movements (e.g., an eye tracker), the perception of the pedestrian was identified at the moment in which the driver started to decrease his/her speed, releasing the accelerator pedal. This is a usual practice in such studies [33,34,36,41].

**Figure 3:**
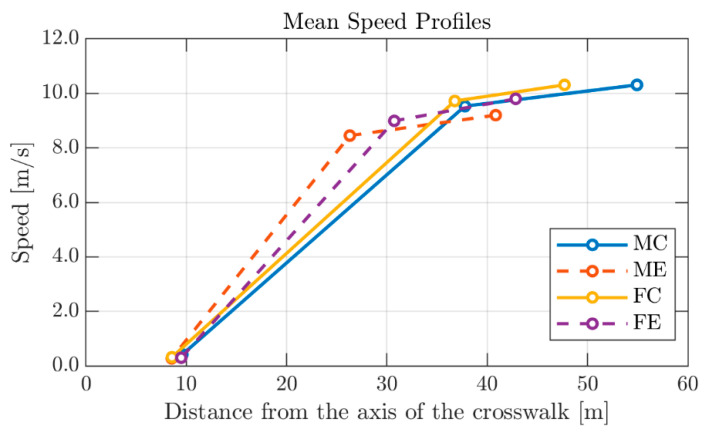
Drivers’ mean speed profiles. *Legend*: MC: male drivers in the control group; ME: male drivers in the experimental group; FC: female drivers in the control group; FE: female drivers in the experimental group.

The braking behavior of the drivers’ cohort was characterized looking at their speed profiles along a section of 100 m in advance the crosswalk [30] and collecting the operational indicators suggested by the literature [33,34,42]:

- $V_{i}$ and $L_{Vi}$: the driver’s speed (or “initial speed”) and associated distance from the crosswalk when (s)he decided to release the accelerator pedal and decrease the vehicle speed after perceiving the pedestrian on the sidewalk;
- $TTZ_{d}=L_{Vi}/V_{i}$: the expected time for the driver to reach the crossing pedestrian if (s)he continues driving at the same initial speed $V_{i}$;
- $V_{b}$ and $L_{Vb}$: the driver’s speed and associated distance from the axis of the pedestrian crossing when (s)he applied the brakes;
- $L_{Vmin}$: the distance from the conflict point at which the vehicle’s minimum speed $V_{min}$ has been observed;
- $\sigma \left(V_{n}\right)$: the standard deviation of vehicle speed during the braking maneuver, also called fluctuation in speed [42];

It is worth noting that the simulated scenario ($TTZ_{p}=3\;s$) necessarily forced the driver to stop or to drastically reduce the vehicle’s initial speed. For this reason, the minimum speed of the braking maneuver was not considered among the study variables. Figure 3 shows the drivers’ mean speed profiles (sketched by means of $V_{i}$ and $L_{Vi}$, $V_{b}$ and $L_{Vb}$, $V_{min}$ and $L_{Vmin}$) for each of the four groups considered in the study: MC—male drivers in the control group, ME—male drivers in the experimental group, FC—female drivers in the control group, FE – female drivers in the experimental group. All features parameters of participants’ braking maneuvers are reported in the supplementary material (Table S1).

The selected variables were analyzed using a mixed two-way ANOVA with Group (1. Control Group; 2. Experimental Group) and Gender (1. Male drivers; 2. Female drivers) as between-subject factors, and the driving parameters as dependent variables ($TTZ_{d}$, $V_{i}$, $L_{Vi}$,$V_{b}$, $L_{Vb}$, $L_{Vmin}$, $\sigma \left(V_{n}\right)$). The application of the ANOVA test involves the verification of some basic assumptions: the absence of outliers, the normality of the dependent variables and the homogeneity of the variance among the groups. In particular, the box-plots did not identify any outliers among the selected variables, whose normality was assessed by means of the Shapiro-Wilk test (*p* > 0.05). Except for the $L_{Vb}$ parameter associated with the group of male drivers in the experimental group (group ME), all other dependent variables met the assumption of a normal distribution of scores (Table 2) for each of the four defined groups. Anyway, the sample size (the group ME consists of 30 drivers) and the robustness of the ANOVA test against the violation of this assumption [43] made possible the application of such a method also for the $L_{Vb}$ variable. Finally, the Levene’s test (*p* > 0.05) showed that the variance of each dependent variable between groups was equal (last row of Table 2). The significance of Group or Gender effect was evaluated using unweighted means and Type III sums of squares [44].

Other parameters of interest characterizing participants’ braking maneuvers were the average deceleration rate (the average deceleration to pass from $V_{i}$ to $V_{min}$) [33,34,45] and fluctuations in position [42], for example with respect to the center of the lane (i.e., the standard deviations of the distance between the center of the lane and that of the vehicle). However, none of the variables listed above met the requirements for applicability of the ANOVA analysis, neither in terms of normality nor homogeneity of variance among the groups; for this reason, they were not considered in the following discussion.

## 4. Results and Discussions

Table 3 reports the mean values of the selected dependent variables of the drivers’ braking behavior. It is worth noting that drivers took, on average, similar time to pass from the initial speed to the minimum one: an independent samples t-test was conducted to compare the speed reduction time (or SRT, the time to pass from $V_{i}$ to $V_{min}$) between distracted and non-distracted drivers. There was no significant difference in scores (t(39.02) = −1.57, p = 0.13) between the control group (9.86 s) and the experimental one (8.42 s). This result shows that participants yielded to the pedestrian well before she started to cross (SRT>>6 s, [36,45]).

### 4.1. The Actual Time Left for the Vehicle to Arrive at the Conflict Point

**Figure 4:**
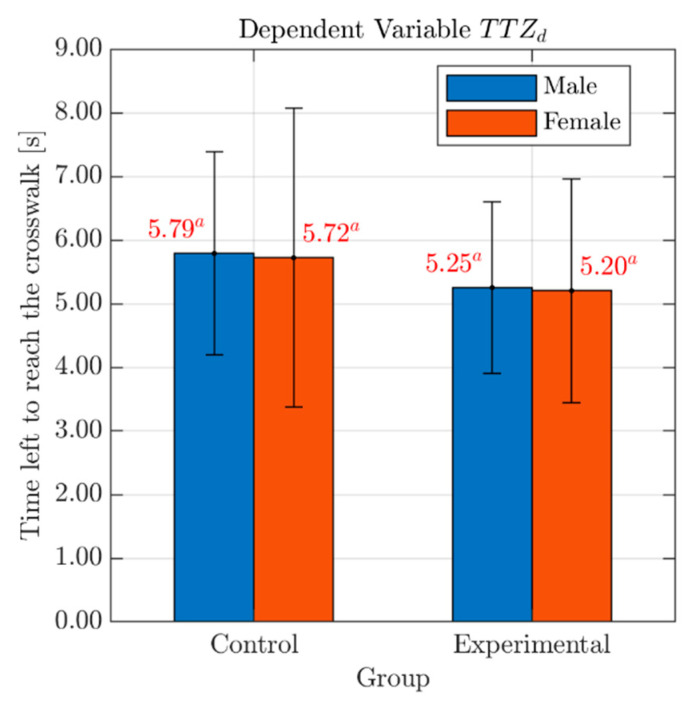
Effects of Gender and Group on the variable $TTZ_{d}$. *Legend*: Red numbers above the bars represent mean values of the variable $TTZ_{d}$, for males (blue bar) and females (red bar) in each Group. Statistically significant differences are indicated by different superscript letters. Standard deviation error bars are also reported for each sub-group.

As for the actual condition of the vehicle–pedestrian interaction that occurred during the tests ($TTZ_{d}$; see Table 3), the analysis showed the absence of any significant Group-related difference (F(1, 74) = 1.67, p = 0.20) with no effects of Gender (F(1, 74) = 0.02, p = 0.89) nor Group*Gender interaction (F(1, 74) = 0.00, p = 0.98). Bar graph with mean values and standard deviation errors of the variable $TTZ_{d}$, for both males and females in each Group, is presented in Figure 4.

The mean value of $TTZ_{d}$ for the two groups was similar (Control group: 5.76 s; Experimental group: 5.23 s, see Table 3). This result confirmed what was previously observed for the speed reduction time: it showed that the pedestrian has a sufficient safety margin to pass before the car and that such a driving behavior ($TTZ_{d}>4\;s$) does not influence the safety of the pedestrian [31]. These findings add evidence to a growing body of research on cognitive distraction, suggesting that conversing on a hands-free mobile phone while driving may have no associated increase in collision risk [22] and, in particular for this study, no increase in pedestrian-safety risk. However, such condition does not exclude the potential presence of detrimental driving performance effects, particularly of compensatory nature, as can be verified in the existing literature [46,47]. For an exhaustive description of potential reasons why findings of impaired driver performance may not result in a corresponding increase in crash risk, please refer to Dingus et al. [22].

Therefore, it is possible to proceed to a comparison (under equal pedestrian-safety risk) among participants’ speed profiles with the aim to detect the presence of significant effects of cognitive distraction on driving skills and, consequently, to validate story retelling as an effective method to increase mental workload in a driving simulation test. In order to carry out such comparison of speed profiles, vehicles’ speed and distance from the crosswalk were analyzed separately, as already performed by Bella et al. [33,34,47] and Saifuzzaman et al. [42].

### 4.2. Driver’s Initial Speed and Associated Distance from the Conflict Point

**Figure 5:**
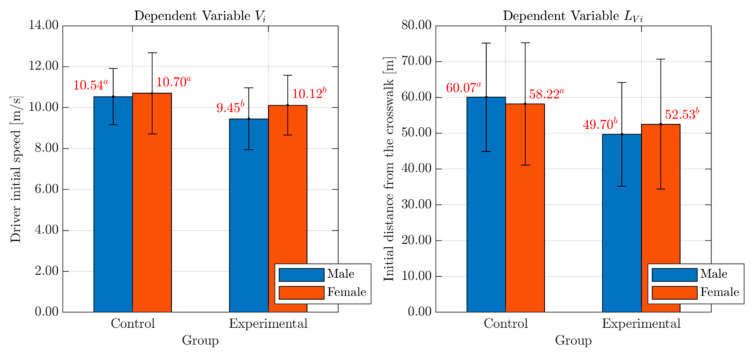
Effects of Gender and Group on the variables $V_{i}$ and $L_{Vi}$. *Legend*: Red numbers above the bars represent mean values of the variables $V_{i}$ and $L_{Vi}$, for males (blue bar) and females (red bar) in each Group. Statistically significant differences are indicated by different superscript letters. Standard deviation error bars are also reported for each sub-group.

As for the drivers’ initial speed ($V_{i}$; see Table 3), the analysis showed the presence of a significant Group-related difference with a medium effect size (according to Cohen’s criterion [48]): (F(1, 74) = 4.94, p < 0.03, partial eta squared = 0.06). Interestingly, the analyses revealed the absence of any effect of gender (F(1, 74) = 1.21, p = 0.28) with no significant Group*Gender interaction (F(1, 74) = 0.46, p = 0.50). Similarly, also on the associated distance from the conflict point ($L_{Vi}$, see Table 3), the Group-related difference was significant with a medium effect size (F(1, 74) = 4.22, p < 0.04; partial eta squared = 0.05) and no effects of Gender (F(1, 74) = 0.02, p = 0.90) or Group*Gender interaction (F(1, 74) = 0.36, p = 0.55). Bar graphs with mean values and standard deviation errors of the variables $V_{i}$ and $L_{Vi}$, for both males and females in each Group, are presented in Figure 5.

Overall, these results suggest that distracted drivers (both males and females) tend to proceed with a lower speed (9.73 < 10.71 m/s, Table 3) than non-distracted ones, but to begin the braking operation significantly later than controls (50.90 < 59.28 m, Table 3). The effects on initial speed could be interpreted as a compensatory effort [27] for the reduction of cognitive resources allocated to the driving experience [49]. In addition, the mean $L_{Vi}$ positions suggest that the engagement in the cognitive task induced a delayed perception of the pedestrian on the sidewalk [29], as the point in which the driver started to slow down was farther from the crosswalk point for the control group (59.28 m, Table 3) compared to the experimental one (50.90 m, Table 3).

### 4.3. Driver’s Speed at Application of the Brakes and Associated Distance from the Conflict Point

**Figure 6:**
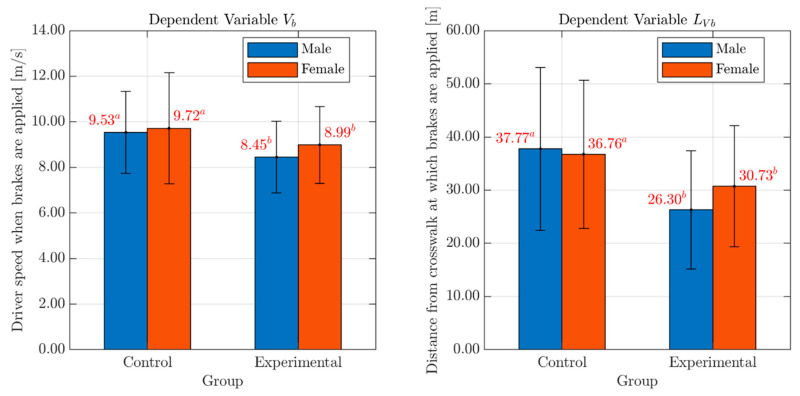
Effects of Gender and Group on the variables $V_{b}$ and $L_{Vb}$. *Legend*: Red numbers above the bars represent mean values of the variables $V_{b}$ and $L_{Vb}$, for males (blue bar) and females (red bar) in each Group. Statistically significant differences are indicated by different superscript letters. Standard deviation error bars are also reported for each sub-group.

As for the drivers’ speed at application of the brakes ($V_{b}$; see Table 3) and associated distance from the conflict point ($L_{Vb}$; see Table 3), the analyses showed the presence of a significant Group-related difference with a medium effect size for $V_{b}$ and a large one for $L_{Vb}$ ($V_{b}$: F(1, 74) = 4.38, p < 0.04; partial eta squared = 0.06; $L_{Vb}$: F(1, 74) = 8.33, p < 0.01; partial eta squared = 0.10). In either case, no effects of Gender ($V_{b}$: F(1, 74) = 0.69, p = 0.41; $L_{Vb}$: F(1, 74) = 0.32, p = 0.58) or Group*Gender interaction ($V_{b}$: F(1, 74) = 0.17, p = 0.68; $L_{Vb}$: F(1, 74) = 0.80, p = 0.37) were found. Bar graphs with mean values and standard deviation errors of the variables $V_{b}$ and $L_{Vb}$, for both males and females in each Group, are presented in Figure 6.

These results confirmed what was previously observed for the variables $V_{i}$ and $L_{Vi}$, showing that the distraction negatively affects the speed profile, at least in the first phase.

### 4.4. Distance from the Conflict Point at the End of the Braking Maneuver

**Figure 7:**
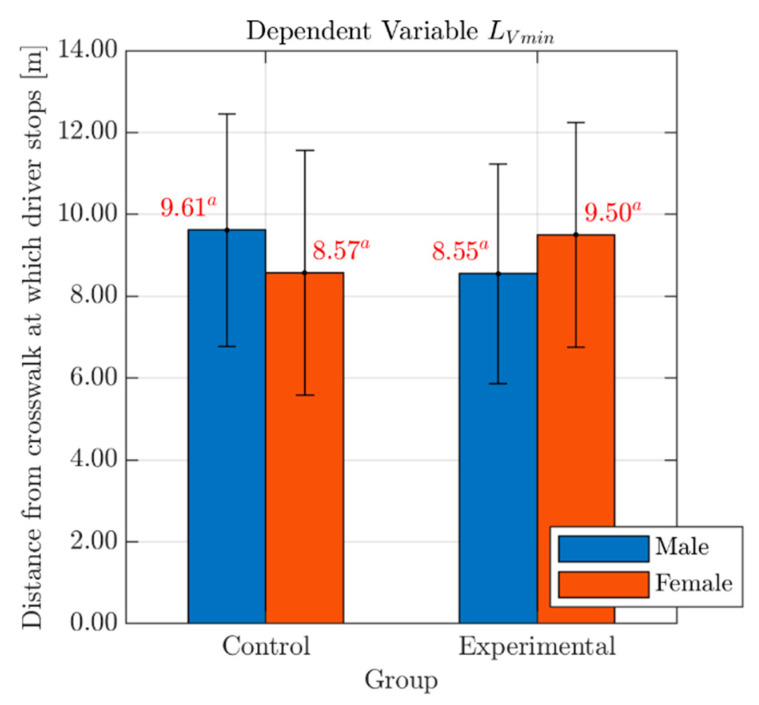
Effects of Gender and Group on the variable $L_{Vmin}$. *Legend*: Red numbers above the bars represent mean values of the variable $L_{Vmin}$, for males (blue bar) and females (red bar) in each Group. Statistically significant differences are indicated by different superscript letters. Standard deviation error bars are also reported for each sub-group.

As for the distance from the conflict point at the end of the braking maneuver ($L_{Vmin}$; see Table 3), the analysis showed the absence of any significant Group-related difference (F(1, 74) = 0.01, p = 0.92) with no effects of Gender (F(1, 74) = 0.00, p = 0.95) or Group*Gender interaction (F(1, 74) = 2.20, p = 0.14). Bar graphs with mean values and standard deviation errors of the variable $L_{Vmin}$, for both males and females in each Group, are presented in Figure 7.

This outcome suggests that, even if effects of the cognitive distraction can be noted in the first phases of the yielding maneuver, the risk-compensating behavior of the drivers in the experimental group may be effective in stopping the vehicle at a distance from the crosswalk comparable to that of participants in the control group. Such a result further confirms that pedestrian safety has not been compromised.

The findings in terms of the drivers’ initial and final positions lead to the obvious conclusion that stopping distances were different across the groups: a two-way between-groups analysis of variance on braking distance showed the presence of a significant Group-related difference with a medium effect size (F(1, 74) = 4.01, p = 0.05, partial eta squared = 0.05). No effects of Gender (F(1, 74) = 0.02, p = 0.89) or Group*Gender interaction (F(1, 74) = 0.11, p = 0.74) was found. In fact, the mean value of the braking distance for distracted drivers (41.90 m) was smaller than that for non-distracted drivers (50.10 m).

### 4.5. Fluctuation in Speed

**Figure 8:**
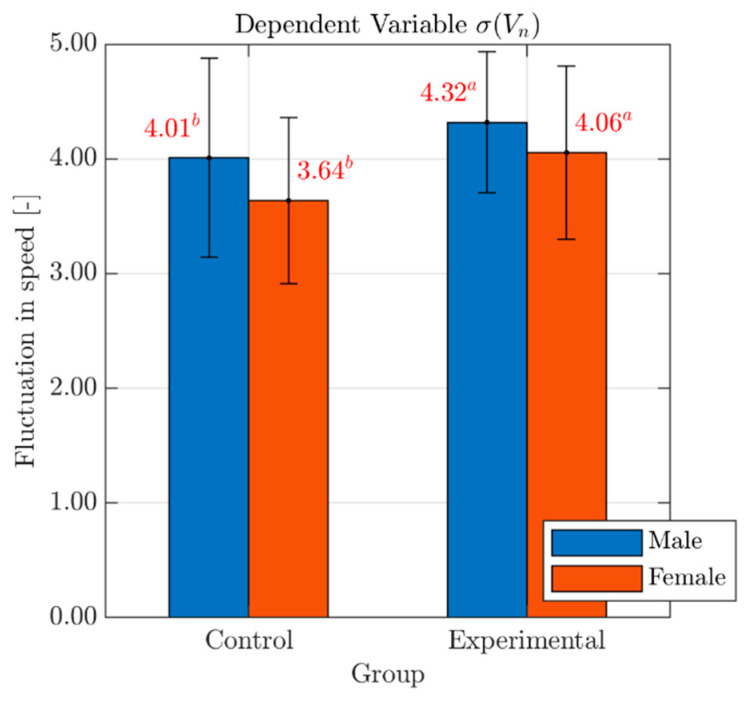
Effects of Gender and Group on the variable $\sigma \left(V_{n}\right)$. *Legend*: Red numbers above the bars represent mean values of the variable $\sigma \left(V_{n}\right)$, for males (blue bar) and females (red bar) in each Group. Statistically significant differences are indicated by different superscript letters. Standard deviation error bars are also reported for each sub-group.

Bar graphs with mean values and standard deviation errors of the variable $\sigma \left(V_{n}\right)$, for both males and females in each Group, are presented in Figure 8. A significant main effect of mobile phone distraction on fluctuation in speed was observed with a medium effect size (F(1, 74) = 4.29, p < 0.04; partial eta squared = 0.06) but no Gender (F(1, 74) = 3.25, p = 0.08) or Gender*Group interaction (F(1, 74) = 0.09, p = 0.76). This suggests that $\sigma \left(V_{n}\right)$ increases with the engagement in the planned cognitive task as distracted drivers may find it difficult to keep speed variations under control. For example (see Table 3), the fluctuation in speed for drivers in the experimental group was 7.1% higher than that for participants in the control group.

## 5. Conclusions

This study compared the braking maneuvers of drivers distracted by a planned hands-free mobile phone conversation (experimental group) with those of un-distracted drivers (control group). Driving data from a cohort of 78 young drivers, aged 20–30 years old, were collected using a virtual car driving simulator. Immersed in a simulated urban scenario, participants were required to respond to an ordinary traffic event: a pedestrian entering a zebra crossing from a sidewalk. The phone call was planned to diminish the amount of cognitive resources allocated to the driving experience. The results of the statistical analyses showed that hands-free mobile phone conversations significantly affected the yielding maneuver. In particular, the effect was statistically significant on speed selection and fluctuation, and on distances from the crosswalk at which the driver released the accelerator pedal or applied the brakes. In fact, the drivers in the experimental group maintained lower speeds compared to baseline drivers, who were left free to drive without any imposed cognitive task. This finding could reflect compensatory behavior for the increased risk associated with the mobile phone conversations, even when earphones are used. Such risk-compensatory behavior has been elsewhere observed and reported in the literature [27,50]. The increase of fluctuation in speed suggests that mobile phone distraction impairs speed control while coping with pedestrians crossing the road [42]. Furthermore, the distances kept by the two groups from the crosswalk in two different moments of the operation (i.e., $L_{Vi}$ and $L_{Vb}$) suggest that distracted drivers perceived the pedestrian on the sidewalk later than baseline drivers. This delayed their braking response, which happened much closer to the conflict point [34]. Differently, no difference was found between behavior of male or female drivers, who seem to have the same affected performance while approaching the zebra crossing.

Based on the foregoing findings, there is sufficient evidence that story retelling is effective in reproducing the effects of cognitive distraction on driving performance in a car simulation experiment. This result is not trivial because, at the moment, a protocol or a procedure has not yet been definitively identified for the execution of simulated driving studies aimed at evaluating the driving performance affected by the engagement in a cognitive secondary task. Therefore, the procedure presented in this study, designed primarily from a psychological point of view, is a useful reference for researchers studying driving behavior, affected by distraction, in response to new road-design solutions or specific traffic events.

This study contributes to the growing evidence that hands-free mobile phone use while driving significantly affects driving performance [27,29,46,47,49]. However, further assessments on a wider cohort of drivers are needed, not only to confirm the conclusions of this study, but also to investigate and compare the effect of the planned cognitive distraction on different groups, such as younger and older drivers, also considering behavioral components like aggressiveness. In addition, the narrative production skills in participants engaged in the simulated driving task will be addressed in future studies.

## Figures and Tables

**Table 1:** Frequency of Mobile Phone Use While Driving (FMPUWD).

Driver characteristics	Count	Percentage
Mobile phone use while driving:		
Yes	56	71.8
No	22	28.2
FMPUWD to make or take calls:		
At least once in a day (frequent)	16	28.6
Once or twice in a week (moderate freq.)	22	39.3
Once or twice in a month or year (less freq.)	16	28.6
Never	2	3.6
Average time spent on mobile phones while driving (for calls):		
<1 min	27	48.2
2–5 min	23	41.1
6–10 min	4	7.1
>10 min	2	3.6
Wireless earphones or speakerphone usage (for calls):		
Always	34	60.7
Sometimes	18	32.1
Never (handheld use)	4	7.1
FMPUWD to read or write text messages:		
At least once in a day (frequent)	14	25.0
Once or twice in a week (moderate freq.)	12	21.4
Once or twice in a month or year (less freq.)	14	25.0
Never	16	28.6
FMUPWD to read e-mails or surf the internet:		
At least once in a day (frequent)	3	5.4
Once or twice in a week (moderate freq.)	6	10.7
Once or twice in a month or year (less freq.)	3	5.4
Never	44	78.6

**Table 2:** Checking ANOVA test assumptions. *Legend:* the first four rows report the result of the Shapiro-Wilk normality test (*p*-values) for each of the four groups considered in the study (i.e., FE, FC, ME, MC) and separately for each variable of the speed profile (i.e., $TTZ_{d}$, $V_{i}$, $L_{Vi}$, $V_{b}$, $L_{Vb}$, $L_{Vmin}$, $\sigma \left(V_{n}\right)$); the last row reports the result (*p*-values) of the variance homogeneity test (or Levene’s test) that checks whether the variance in scores is the same for each of the four groups, separately for each variable of the speed profile.

Groups	Tests	*p*-Value Results (Significance Value at the *p* > 0.05 Level)						
		$TTZ_{p}$	$V_{i}$	$L_{Vi}$	$V_{b}$	$L_{Vb}$	$L_{Vmin}$	$\sigma \left(V_{n}\right)$
FE	Shapiro-Wilk	0.505	0.545	0.888	0.867	0.950	0.944	0.178
FC	Shapiro-Wilk	0.936	0.956	0.502	0.981	0.565	0.143	0.379
ME	Shapiro-Wilk	0.835	0.183	0.130	0.159	0.000	0.208	0.727
MC	Shapiro-Wilk	0.144	0.921	0.225	0.759	0.851	0.359	0.198
ALL	Levene	0.192	0.511	0.812	0.463	0.463	0.991	0.294

**Table 3:** Mean value (standard deviation) of the speed profile variables across groups. *Legend*: $TTZ_{d}$ is the time left for the vehicle to reach the crosswalk; $V_{i}$ and $L_{Vi}$ are speed and associated distance from the crosswalk when the driver released the accelerator pedal; $V_{b}$ and $L_{Vb}$ are speed and associated distance from the crosswalk when the driver applied the brakes; $L_{Vmin}$ is the distance from the crosswalk at which the minimum speed was observed; $\sigma \left(V_{n}\right)$ is the standard deviation of vehicle speeds.

Groups	$TTZ_{p}\;(s)$	$V_{i}\;(m/s)$	$L_{Vi}\;(m)$	$V_{b}\;(m/s)$	$L_{Vb}\;(m)$	$L_{Vmin}\;(m)$	$\sigma \left(V_{n}\right)$ (-)
Control	5.76 ^a^ (1.91)	10.61 ^a^ (1.62)	59.28 ^a^ (15.68)	9.61 ^a^ (2.05)	37.35 ^a^ (14.46)	9.17 ^a^ (2.89)	3.85 ^b^ (0.82)
Experimental	5.23 ^a^ (1.52)	9.73 ^b^ (1.51)	50.90 ^b^ (16.01)	8.67 ^b^ (1.63)	28.17 ^b^ (11.35)	8.95 ^a^ (2.72)	4.21 ^a^ (0.68)

*Note:* Different superscript letters indicate statistically significant differences between Groups (*p* < 0.05).

## Supplementary Materials

The following table is available online at https://www.mdpi.com/2076-328X/10/10/150/s1, Table S1: Features parameters of the participants’ braking maneuvers.
